# Effects of Neoadjuvant Radiotherapy on Postoperative Complications in Rectal Cancer: A Meta-Analysis

**DOI:** 10.1155/2022/8197701

**Published:** 2022-01-05

**Authors:** Jianguo Yang, Yajun Luo, Tingting Tian, Peng Dong, Zhongxue Fu

**Affiliations:** ^1^Department of Gastrointestinal Surgery, The First Affiliated Hospital of Chongqing Medical University, Chongqing 400016, China; ^2^Department of Gastroenterology, Zhutuo Town Health Center of Chongqing, Chongqing 402191, China

## Abstract

**Objective:**

Neoadjuvant radiotherapy (nRT) is an important treatment approach for rectal cancer. The relationship, however, between nRT and postoperative complications is still controversial. Here, we conducted a meta-analysis to evaluate such concerns.

**Methods:**

The electronic literature from 1983 to 2021 was searched in PubMed, Embase, and Web of Science. Postoperative complications after nRT were included in the meta-analysis. The pooled odds ratio (OR) was calculated by the random-effects model. Statistical analysis was conducted by Review Manager 5.3 and STATA 14.

**Results:**

A total of 23,723 patients from 49 studies were included in the meta-analysis. The pooled results showed that nRT increased the risk of anastomotic leakage (AL) compared to upfront surgery (OR = 1.23; 95% CI, 1.07–1.41; *p*=0.004). Subgroup analysis suggested that both long-course (OR = 1.20, 95% CI 1.03–1.40; *p*=0.02) and short-course radiotherapy (OR = 1.25, 95% CI, 1.02–1.53; *p*=0.04) increased the incidence of AL. In addition, nRT was the main risk factor for wound infection and pelvic abscess. The pooled data in randomized controlled trials, however, indicated that nRT was not associated with AL (OR = 1.01; 95% CI 0.82–1.26; *p*=0.91).

**Conclusions:**

nRT may increase the risk of AL, wound infection, and pelvic abscess compared to upfront surgery among patients with rectal cancer.

## 1. Introduction

Colorectal cancer (CRC) is a common malignant tumor globally that is ranked third in terms of incidence and second in terms of mortality. It is estimated that over 1.8 million new cases of CRC occur each year [1]. In the past few decades, the development of total mesorectal excision (TME) has greatly improved the oncological outcome of rectal cancer patients. However, local recurrence, distant metastasis, and chemoradiotherapy resistance are still the main causes of death in rectal cancer patients [2, 3]. Studies have shown that preoperative chemoradiotherapy downstages the primary tumor, increases the possibility of radical resection, increases the sphincter-preserving rate, and reduces the risk of local recurrence of rectal cancer [4–7]. Therefore, TME after neoadjuvant chemoradiotherapy (nCRT) has become the standard treatment for locally advanced rectal cancer. After nCRT or total neoadjuvant therapy (TNT), approximately 15–30% of rectal cancer patients can achieve pathological complete response (PCR), which significantly improves the oncological outcome of patients [8–10].

Postoperative complications are closely related to local recurrence and distant metastasis of rectal cancer [11]. AL is one of the common postoperative complications of rectal cancer. It has been reported that AL increases the risk of systemic, peritoneal, and local recurrence of CRC [12, 13], and the possible mechanism is that the inflammatory reaction results in an increase in proinflammatory and proangiogenic factors, which may stimulate the growth of residual tumor cells. In addition, inflammation caused by AL contributes to tumor escape immune surveillance by suppressing T cell [14, 15]. Several meta-analyses have shown that AL decreases overall survival and disease-free survival and increases the risk of cancer-related death in rectal cancer patients [16–18]. Many factors may affect postoperative complications, including age, sex, tumor location, and diabetes mellitus [19–21]. Some studies have reported that chemoradiotherapy may create local rectal tissue injury and influence anastomosis healing. However, it remains controversial whether preoperative chemoradiotherapy leads to an increase in complications after rectal cancer surgery [22–24].

We conducted the present meta-analysis to explore whether nRT increases the risk of postoperative complications for rectal cancer. The primary outcome of interest was AL, and the secondary outcomes of interest included wound infection, pelvic abscess, urinary tract infection, ileus, hemorrhage, reoperation, overall complications, and mortality.

## 2. Methods

The systematic review and meta-analysis were performed based on the preferred reporting items for the systematic review and meta-analysis 2020 statement (PRISMA 2020 statement) [25] (Table S1).

### 2.1. Literature Search Strategy

PubMed, EMBASE, and Web of Science electronic databases were searched by two authors (Yang and Luo) with the following subject terms: (rectal cancer) OR (rectal neoplasms); (neoadjuvant therapy) OR (neoadjuvant radiotherapy) OR (neoadjuvant chemoradiotherapy) OR (perioperative therapy) OR (perioperative radiotherapy) OR (perioperative chemoradiotherapy); and (complications) OR (morbidity) OR (anastomotic leakage) OR (anastomotic leak). We combined the search items using “AND”. The last data retrieval was June 1, 2021, and language restrictions were not considered. The reference lists of selected articles were searched to find potentially relevant studies. Titles, abstracts, and full texts of studies were accessed to exclude inappropriate research. When several articles were published in the same cohort, only the latest publications were included. If there were inconsistent decisions, they were resolved by two reviewers through consultation. Otherwise, the final decision was made by a third reviewer (Fu).

### 2.2. Study Selection Criteria

The inclusion criteria were as follows: (1) cohort studies and randomized clinical trials (RCTs); (2) English publication studies; (3) postoperative complications in the nRT group and upfront surgery group were compared; and (4) AL must be reported in eligible studies. The exclusion criteria were as follows: (1) reviews, letters, expert opinions, comments, case reports, and meta-analysis; (2) incomplete data (no primary outcome or detailed data of postoperative complications) or no full text; (3) nonhuman studies; and (4) nonrelevant literature.

### 2.3. Data Extraction and Quality Assessment

All data were extracted from full texts by two authors (Yang and Tian) with a standard spreadsheet. The collection information was as follows: (1) first author name, journal name, publication time, nation, and the number of participants; (2) basic characteristics and therapy process of rectal cancer patients; and (3) postoperative complications (AL, wound infection, pelvic abscess, urinary tract infection, ileus, hemorrhage, reoperation, and overall complications) and mortality.

The quality of cohort studies was evaluated independently according to the Newcastle–Ottawa Scale (NOS) by two reviewers (Luo and Peng). The NOS Scale of cohort study included study population option, comparability, and exposure or outcome assessment. The maximum score was 9 points for cohort studies, and studies with a score of 6 or more were considered high quality [26]. Two researchers used the Cochrane risk-of-bias tool (RoB 2.0) to independently assess the quality of randomized controlled trials. The study was assigned an overall score: “low,” “some concerns,” or “high.” [27]. Any disagreement issues were resolved through negotiating with each other or consulting with a third reviewer.

#### 2.3.1. Definition

The diagnosis of AL was required to meet at least one of the following conditions: (1) intestinal contents and/or gas leakage into the abdomen or pelvis from the anastomotic site and extravasated through the wound, drainage tube, or anus; (2) postoperative recurrent fever, abdominal pain, sepsis, peritonitis and/or organ failure; and (3) confirmed by imaging examination (such as X-ray, endoscopy, computed tomography, magnetic resonance imaging, or ultrasound) or digital rectal examination or surgery [28]. AL that was only detected by imaging but had no clinical manifestations was defined as “asymptomatic” AL. We only pooled symptomatic AL data in the present meta-analysis. Wound infection included abdominal and perineal wound infection. Overall complications included surgical and nonsurgical complications within 30 days after surgery. Mortality was defined as death within 30 days after surgery or during hospitalization. Neoadjuvant radiotherapy (nRT) included short-course radiotherapy (SRT), long-course radiotherapy (LRT), and chemoradiotherapy (CRT).

### 2.4. Statistical Analysis

Statistical analysis was conducted by Review Manager version 5.3 (The Nordic Cochrane Center, The Cochrane Collaboration, Copenhagen, Denmark) and STATA 14 (Stata Corporation, College Station, TX, USA). Dichotomous data were summarized as odds ratios (ORs) with 95% confidence intervals (CIs) using the Mantel–Haenszel method [29]. Due to the expected heterogeneity between studies, the random-effects model was applied to all outcomes [30]. Heterogeneity was evaluated by the I^2^ index and *Q* test. I^2^ values of <25%, 25–50%, 50–75%, and 75–100% suggest low, moderate, high, and extreme heterogeneity, respectively [29]. *I*^2^ ≥ 50% or *p* < 0.01 (Cochran's *Q* test) was considered significant heterogeneity. If there was significant heterogeneity in the pooled data, subgroup analysis was used to assess the potential reason for the heterogeneity [31]. Publication bias was evaluated by visual inspection of the funnel plot and Egger's test, providing that more than 10 studies with the primary outcome of interest were included [32]. The pooled OR and 95% CI were represented by the forest plot. *p* values less than 0.05 were considered significant.

In addition, we conducted a subgroup analysis of anastomotic leakage. The purpose was to explore the effect of neoadjuvant long-course radiotherapy, short-course radiotherapy, and the operation interval (<8 weeks) after long-course radiotherapy on anastomotic leakage. We also performed a subgroup analysis of RCT and non-RCT studies.

## 3. Results

### 3.1. Literature Search and Characteristics

**Figure 1:**
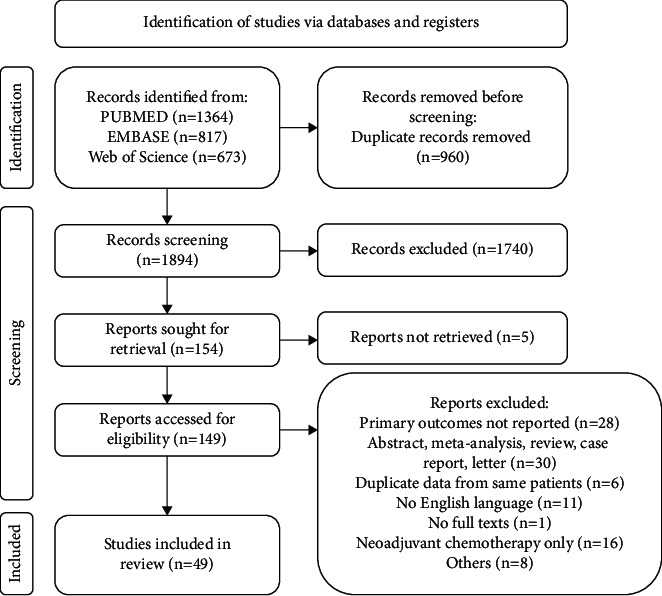
PRISMA flow diagram.

According to the search strategy, 2854 articles were retrieved. After removing 960 duplicate documents, 1894 potentially related studies were included. A total of 1740 articles were excluded by reading the titles and abstracts. After reading the full text, the following 100 studies were excluded: results of primary interest not reported (*n* = 28); abstract, meta-analysis, reviews, case reports, and letters (*n* = 30); duplicate data from same patients (*n* = 6); non-English language text (*n* = 11); no full text (*n* = 1); neoadjuvant chemotherapy only (*n* = 16); others (*n* = 8). Finally, 10 RCTs, 1 prospective study, and 38 retrospective studies were included in the present meta-analysis (Figure 1).

In total, 23,723 individuals from 49 studies were included in the meta-analysis. Of these individuals, 12,082 received nRT, and 19,502 underwent primary anastomosis after rectal cancer surgery. Eleven of the 49 studies reported SRT with a scheme of 5 Gy each time for 5 consecutive days. Radical operation was conducted within 1 week after the completion of SRT. The majority of CRT was combined with fluorouracil-based concurrent chemotherapy, and the concurrent chemotherapy regimens mainly included 5FU, 5FU + LV, and capecitabine. The specific characteristics of each study are shown in Table 1 and Table S2.

### 3.2. Assessment of Methodological Quality and Validity

The quality of the cohort study was assessed by the NOS scale. The results showed that the bias of the included studies was acceptable, because all studies received a score of five stars or above. We assessed the risks of bias of the 10 included RCTs using Cochrane RoB 2.0. Six RCTs were considered to be at low risk of bias. Four RCTs had some concerns because of the randomization process or deviation from the intended intervention (Table S3 and Figure S7).

### 3.3. Anastomotic Leakage

**Figure 2:**
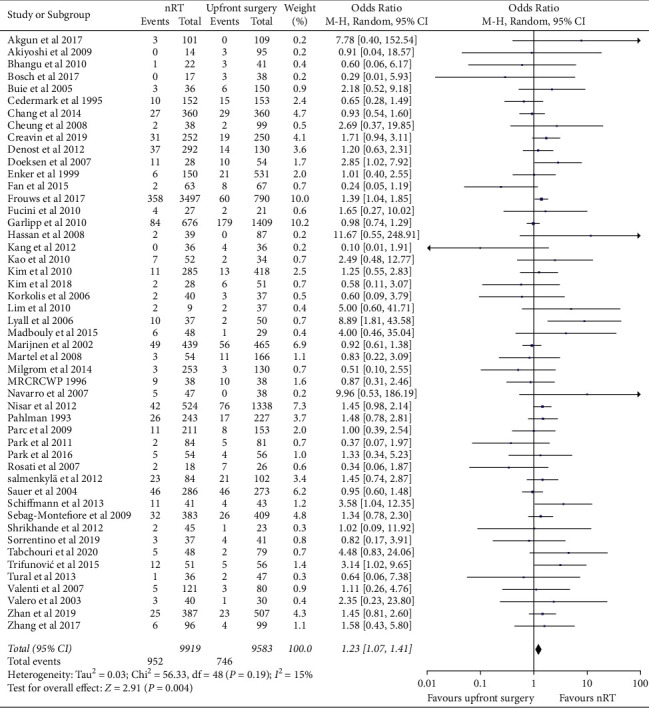
Impact of neoadjuvant radiotherapy on anastomotic leakage.

For the primary endpoint of AL, 49 studies were analyzed. A total of 19,502 patients underwent anterior resection, including 9919 patients from the nRT group. The pooled OR of AL was 1.23 (95% CI, 1.07–1.41; *p*=0.004) (Figure 2). There was low heterogeneity among these studies (*χ*^2^ = 56.33, *p*=0.19; *I*^*2*^ = 15%).

**Figure 3:**
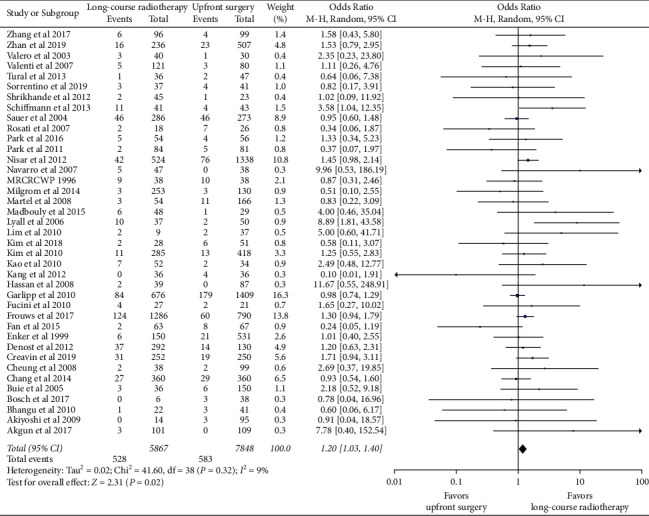
Subgroup analysis: the effect of neoadjuvant long-course radiotherapy on anastomotic leakage.

We conducted a subgroup analysis for AL. For one subgroup analysis, 39 studies reported the incidence of AL in patients with long-course radiotherapy (including CRT) before surgery. The pooled data showed that the risk of AL in patients with long-course radiotherapy was higher than that in patients without long-course radiotherapy (OR = 1.20, 95% CI, 1.03–1.40; *p*=0.02) and no obvious heterogeneity was detected (*χ*^2^ = 41.60, *p*=0.32; *I*^2^ = 9%) (Figure 3). Eleven studies of patients receiving SRT were included in the meta-analysis. The pooled data revealed that SRT also increased the incidence of AL (OR = 1.25, 95% CI, 1.02–1.53; *p*=0.04) with low heterogeneity (*χ*^2^ = 11.30, *p*=0.33; *I*^*2*^ = 11%) (Figure S1).

**Figure 4:**
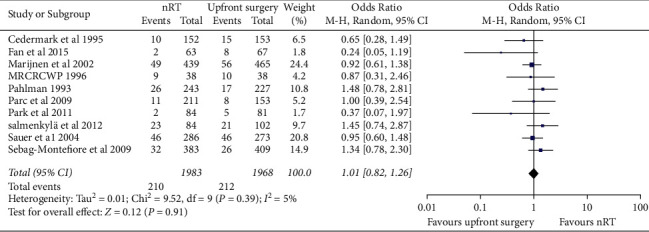
Forest plots of anastomotic leakage after neoadjuvant radiotherapy in randomized controlled trials.

Ten RCTs involving 3951 patients with rectal cancer were assessed. The analysis demonstrated no significant association of AL with nRT (OR = 1.01; 95% CI 0.82–1.26; *p*=0.91), and no heterogeneity was observed (*χ*^2^ = 9.52, *p*=0.39; *I*^*2*^ = 5%) (Figure 4). In contrast, the pooled data in the 39 retrospective studies found that the incidence of AL in the nRT group was higher than that in the upfront surgery group (OR = 1.33; 95% CI 1.13–1.57; *p*=0.0008) (Figure S2).

Twenty-eight studies reported that radical surgery was performed within 8 weeks after the completion of long-course radiotherapy. The pooled results indicated that surgery within eight weeks did not increase the incidence of AL compared to the upfront surgery group (OR = 1.12, 95% CI, 0.94–1.34; *p*=0.20), and there was no significant heterogeneity among the included studies (*χ*^2^ = 28.39,*p*=0.39; *I*^*2*^ = 5%) (Figure S3).

### 3.4. Wound Infection and Pelvic Abscess

**Figure 5:**
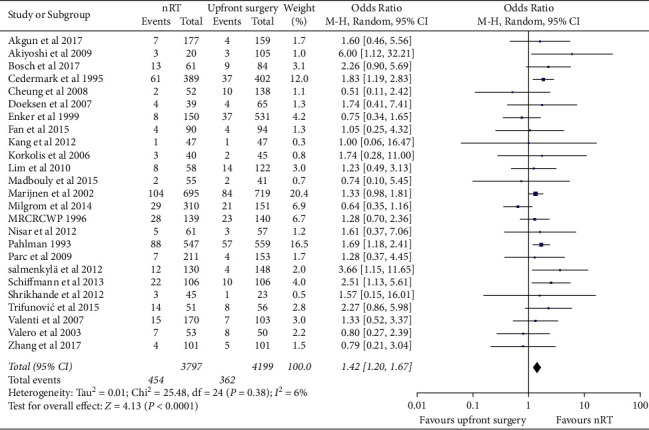
Forest plot of postoperative wound infection in patients with neoadjuvant radiotherapy.

Wound infection data were analyzed in 25 studies, and 12 of these studies reported perineal wound infection after abdominoperineal resection. The pooled data showed that patients receiving nRT were more likely to suffer from wound infection (OR = 1.42, 95% CI, 1.20–1.67; *p* < 0.00001) (Figure 5). In addition, the risk of perineal wound infection after abdominoperineal resection in the nRT group was higher than that in the upfront surgery group (OR = 2.14, 95% CI, 1.72–2.68; *p* < 0.00001), and the heterogeneity was not significantly different (Figure S4). For postoperative pelvic abscess, nRT showed a higher incidence of pelvic abscess than upfront surgery (OR = 2.12, 95% CI, 1.52–2.96; *p* < 0.00001) (Figure S5).

### 3.5. Other Postoperative Complications

Ten studies reported urinary tract infections after surgery. The present study found that nRT did not increase the risk of urinary tract infections (OR = 1.15, 95% CI, 0.77–1.71; *p*=0.41). Fourteen studies described postoperative hemorrhage, and the pooled incidence of postoperative hemorrhage in the nRT group and upfront surgery group was 2.83% and 2.78%, respectively. In addition, nRT did not increase the incidence of postoperative ileus. The pooled data showed that the overall complications in the nRT group were higher than those in the upfront surgery group, but there was no significant difference (OR = 1.15, 95% CI, 0.99–1.32, *p*=0.06). Moderate heterogeneity was observed in the meta-analysis (*χ*^2^ = 37.47, *p*=0.06; *I*^*2*^ = 39%). In this meta-analysis, the nRT group and the upfront surgery group had similar reoperation and mortality rates. The specific data on urinary tract infection, hemorrhage, ileus, overall complications, reoperation, and mortality are shown in Table 2.

### 3.6. Publication Bias

The visual funnel plot distribution and Egger's test were used to assess the publication bias of AL. According to the funnel plot and Egger's test results, no publication bias was detected (*p*=0.37, Egger's test) (Figure S6).

## 4. Discussion

The present meta-analysis was designed to investigate the effect of nRT on postoperative complications. The present study suggested that nRT may be related to increased incidence of AL compared to upfront surgery. Moreover, other anticipated results were as follows: nRT also increased the risk of postoperative wound infection and pelvic abscess.

In accordance with the present results, both short-course radiotherapy and long-term radiotherapy increased the incidence of AL within 30 days after surgery. Qin et al. [79] performed a randomized controlled trial and reported similar results to the present study. A total of 260 patients with anterior resection were enrolled in their study, of whom 61.92% received nCRT. The incidence of symptomatic AL within 30 days after surgery was significantly higher than that of neoadjuvant chemotherapy alone (16.15% vs. 5.1%). Additionally, Frouws et al. [46] retrospectively analyzed 3001 rectal cancer patients who underwent primary anastomosis, of whom 2211 patients received SRT. Their study found that the AL rate of Grades B and C in the SRT group was higher than the upfront surgery group (10.58% vs. 7.59%). In contrast, there was no evidence that nRT was a risk factor for AL in multiple large randomized controlled trials. An earlier multicenter randomized controlled study in Sweden indicated that preoperative short-course radiotherapy did not increase the incidence of AL compared to surgery alone (10.97% vs. 7.66%) [63]. The trial conducted by Marijnen et al. [57] also found that the incidence of AL was similar between the short-course radiotherapy and surgery alone groups (11% vs. 12%). Another prospective study reported that short-course radiotherapy had a higher rate of AL than surgery alone (27.4% vs. 20.6%), but the results were not significantly different [68].

Consistent with short-course radiotherapy, a phase III clinical trial performed in Korea showed that, compared with surgery alone, TME surgery after long-course radiotherapy did not result in more AL [65]. The CAO/ARO/AIO-94 study reported that the incidence of AL was similar between the neoadjuvant chemoradiation group and adjuvant chemoradiation groups (11% vs. 12%) [4]. Moreover, the meta-analysis including 10 RCTs also did find nRT would increase the occurrence of AL compared with upfront surgery. Considering that no association between AL and nRT was observed in RCTs, these results need to be interpreted with caution.

In the present meta-analysis, no evidence suggested that surgery within 8 weeks after completing radiotherapy would cause a higher risk of AL. The Lyon R90-01 study results indicated that prolonging the interval between completing radiotherapy and surgery may promote tumor downstaging, improve sphincter preservation, improve PCR rates, and have no influence on postoperative complications and oncological outcomes [80]. In recent years, an interval of 6–8 weeks after radiotherapy has been considered the optimal time for surgery. The results of two previous two meta-analyses have also shown that extending the interval of radiotherapy and surgery to 8 weeks could achieve a higher PCR rate without increasing postoperative complications [81, 82]. Nevertheless, the GRECCAR-6 study found that prolonged operation interval after the end of radiotherapy does not improve the PCR rate and also causes more postoperative complications and surgery time [83]. A possible reason might be that the aggravation of local tissue fibrosis, edema, and inflammatory reaction in the pelvic radiation area change the normal anatomical plane, making it difficult to achieve standard TME surgery and R0 resection. The relationship between PCR and postoperative complications is a debated topic. Maggiori et al. [84] reported that the risk of AL and serious complications is significantly decreased in rectal cancer patients with radiotherapy sensitivity. Another study has also found that patients with PCR have a lower incidence of AL and organ space surgical site infection than patients with non-PCR [85]. In contrast to earlier findings, however, a population-based study has suggested that rectal cancer patients who achieve PCR have higher AL, surgical complications, and all complications [86].

The possible explanation for AL caused by radiotherapy might be that pelvic radiation increases the inflammatory response of the rectum and anastomosis and promotes cicatrization, which affects the healing of the anastomosis [20]. Radiation could also impel fibrosis of the intestinal connective tissue, thereby decreasing the firmness of the anastomosis [87, 88]. In addition, radiotherapy may result in vascular occlusion of the gut and a decrease in vascular density, which hinders the blood supply and venous reflux of the anastomosis [89, 90]. Tissue edema and fibrosis in radiotherapy areas also challenge the surgeon's technique.

It has been reported that defunctioning stoma can decrease the risk of AL and the serious consequences after AL [91–93]. Two meta-analyses have shown that the incidence of AL and reoperation rates in patients with defunctioning stoma are lower than those in patients without a defunctioning stoma [94, 95]. Another meta-analysis involving 23 observational studies further concluded that defunctioning stoma is an important protective factor for AL after low anterior resection [96]. Unfortunately, several retrospective studies have demonstrated that defunctioning stoma does not impact the occurrence of AL but instead decreases the severe clinical symptoms and reoperation rate after AL [97–101]. In the present study, the pooled data from 21 studies showed that the defunctioning stoma rate in the nRT group was significantly higher than that in the upfront surgery group (69.44% vs. 47.39%). Hence, we hypothesized that defunctioning stoma may decrease the occurrence of symptomatic AL after nRT. The main factors of AL after anterior resection are poor blood supply, excessive tension, and poor local condition of the intestine at the anastomosis. Defunctioning stoma directly reduces the pressure in the rectum and the defecation reflex caused by the stimulation of intestinal contents, thus preventing the occurrence of AL. It should be noted that defunctioning stoma did not decrease the risk of AL related to the poor anastomosis blood supply.

The present study also confirmed that nRT was associated with surgical site infection. The Stockholm Phase I trial randomly divided patients into the preoperative radiation therapy group (5 × 5 Gy) and surgery alone group [38]. The results indicated that the risk of wound infection in patients with radiotherapy was higher than that of patients with surgery alone (14% vs. 9%). Three other prospective studies also reported that SRT not only increased the occurrence of wound infection, but was also a risk factor for perineal wound infection and pelvic abscess after APR [6, 57, 63]. Consistent with SRT, CRT also plays an important role in surgical site infection after rectal cancer surgery. El-Gazzaz et al. conducted a retrospective study of 696 patients undergoing APR [102]. The data showed that, compared to surgery alone, patients receiving CRT had a higher percentage of perineal wound infections (18% vs. 11%). A previous meta-analysis has also found that CRT is closely related to wound infection, perineal wound infection, and AL [103]. The possible reasons are that radiation not only damages tumor cells, but also affects the peripheral microcirculation, resulting in obstructive vasculitis and delayed wound healing.

The present meta-analysis may have several limitations. First, most of the included studies were retrospective studies with a wide publication time range. Second, due to the limited number of included studies, some subgroup analysis conclusions may not be reliable. Third, the incidence of symptomatic AL in this meta-analysis ranged from 0% to 39.29%. This large difference in the AL rate may be due to the inconsistent definition of AL and the proportion of patients undergoing anterior rectal resection. Moreover, the conclusion that nRT increases the AL rate must be interpreted carefully. The pooled data in the RCTs showed that patients receiving nRT did not observe a higher AL rate than patients receiving upfront surgery. Fifth, we excluded 11 non-English articles. Although we found that it had no significant impact on the results after including non-English studies, it might also increase the risk of selection bias and reduce reliability. Finally, the chemotherapy regimens were different in the included CRT studies. Studies have reported that different chemotherapy regimens are associated with postoperative complications [104, 105]. However, the present meta-analysis did not conduct a subgroup analysis of the relationship between different chemotherapy regimens and postoperative complications. Hence, more randomized controlled studies are needed to further confirm these results in the future.

## 5. Conclusions

In conclusion, the present study investigated the relationship between nRT and postoperative complications. The meta-analysis suggested that nRT may increase the risk of AL, wound infection, and pelvic abscess compared to upfront surgery among patients with rectal cancer. Moreover, nRT did not significantly affect urinary tract infection, hemorrhage, ileus, reoperation, or mortality.

## Figures and Tables

**Table 1:** The main characteristics of the included studies in the meta-analysis.

Study	Study period	Region	Study type	Men (%)	Tumor location (cm)	TNM stage	Radiation scheme	Radiation dose (Gy/times)	Concurrent chemotherapy	Time to surgery (weeks)	Surgical approach	AL diagnosis	Defunction stoma	No. of patientss
Akgun et al. 2017 [33]	2003–2016	Turkey	PCS/single	40.77	0–15	II–III	CRT	50.4/28	*a* + *b*	4–12	Open	C	Yes	336
Akiyoshi et al. 2009 [34]	2005–2008	Japan	RCS/single	54.40	1–6	II-III	CRT	45/25	*a*	4–8	Lap	NR	Yes	125
Bhangu et al. 2010 [35]	2006–2008	UK	RCS/multi	65.48	NR	I-IV	CRT	30	*a*	6	Lap, Open	C	Yes	84
Bosch et al. 2017 [36]	1991–2010	Netherlands	RCS/multi	64.60	NR	I-IV	SRT/RT/CRT	25/5, 45–50/25–28	*a*	NR	NR	NR	Yes	145
Buie et al. 2005 [37]	1994–2002	Canada	RCS/single	61.38	4–13	I-III	CRT	50.4/25	*a* + *b*	8	NR	*C* ± I	Yes	246
Cedermark et al. 1995 [38]	1980–1987	Swedish	PCS/multi	58.42	NR	I-IV	SRT	25/5	NR	1	NR	NR	NR	791
Chang et al. 2014 [39]	2005–2012	Korea	RCS/single	66.39	NR	I-III	CRT	50.4/28	*a* + *b*, *e* + *f*/*c*	6–8	Lap, Open, Rob	*C* ± I	Yes	720
Cheung et al. 2008 [40]	2000–2007	China	RCS/single	57.37	1–12	I-IV	CRT	50.4/28	*a* + *b*	4–6	Lap	NR	Yes	190
Creavin et al. 2019 [41]	2005–2015	Ireland	RCS/single	NR	NR	II–III	CRT	50–54/25	*a*	10–12	Lap, Open	*C* ± I	Yes	502
Denost et al. 2012 [42]	1999–2010	France	RCS/single	63.01	0–15	I-IV	CRT	45/25	*a* + *b*	6	Lap	C	Yes	422
Doeksen et al. 2007 [43]	2000–2007	Netherlands	RCS/single	56.73	NR	I-IV	SRT	25/5	No	1	NR	*C* ± I	Yes	104
Enker et al. 1999 [44]	1987–1995	USA	RCS/single	59.32	NR	I-III	CRT	50.4/28	*a* + *b*	4–7	NR	*C* ± I	Yes	681
Fan et al. 2015 [45]	2008–2012	China	PCS/single	58.15	0–10	II-III	CRT	46–50/23–25	*c* + *d*	6–10	NR	NR	Yes	184
Frouws et al. 2017 [46]	2009–2013	Netherlands	RCS/multi	60.44	NR	NR	SRT/CRT	25/5, NR	NR	NR	Lap, Open	C	Yes	4287
Fucini et al. 2010 [47]	1997–2000	Italy	RCS/single	58.75	3.7–10.5	II-III	CRT	45/25	*a* + *b*	6–9	NR	NR	NR	80
Garlipp et al. 2010 [48]	2005–2007	Germany	RCS/multi	57.65	0–12	I-III	CRT	50.4/25	a	6	NR	*C* ± I ± *E*	Yes	2085
Hassan et al. 2008 [22]	1982–2001	USA	RCS/single	55.21	1–18	I-IV	LRT	45–53.5/26	NR	6–8	NR	*C* ± I	Yes	126
Kang et al. 2012 [49]	1997–2008	Korea	RCS/single	53.19	1–10	I-III	CRT	50.4–54/28	*a* + *b*	6–8	NR	NR	Yes	94
Kao et al. 2010 [50]	2000–2004	China	RCS/single	57.97	3.49–7.11	II-III	CRT	45–50.4/20–23	*g*+*b*	6–8	NR	NR	Yes	136
Kim et al. 2010 [51]	2000–2006	Korea	RCS/single	65.01	NR	I-IV	CRT	50/25	*a* + *b*/*c*	4–6	NR	C	Yes	703
Kim et al. 2018 [52]	2008–2012	Korea	RCS/single	71.01	4.04–10.19	I-III	CRT	44/22	A/*c*	6–8	Lap, Open, Rob	NR	NR	79
Korkolis et al. 2006 [53]	2000–2004	Greece	RCS/single	64.71	0–15	II-III	SRT	25/5	NR	1	NR	*C* ± I	Yes	85
Lim et al. 2010 [54]	1994–2007	China	RCS/single	58.89	0–4	I-IV	CRT	45–50.4/25	*a*	4–6	Lap, Open	*C* ± I	Yes	180
Lyall et al. 2006 [55]	2001–2006	UK	RCS/single	63.22	NR	II-III	CRT	45/25	*a* + *b*	6–8	NR	*C* ± I	Yes	87
Madbouly et al. 2015 [56]	2009–2012	Egypt	RCS/multi	55.88	NR	II-III	CRT	50.4/28	*a* + *b*	8–11	NR	NR	Yes	96
Marijnen et al. 2002 [57]	1996–1999	Netherlands	PCS/multi	64.36	0–15	I-IV	SRT	25/5	NR	1	NR	*C* ± I	Yes	1414
Martel et al. 2008 [58]	2000–2005	Canada	RCS/single	64.55	NR	NR	CRT	45/25	*A*	6–8	Lap, Open	*C* ± I	Yes	220
Milgrom et al. 2014 [59]	2005–2010	USA	RCS/single	59.44	NR	I-III	CRT	50.4/28	*a*/*c*	4–8	Lap, Open	NR	Yes	461
MRCRCWP. 1996 [60]	1981–1989	UK	PCS/multimulti	68.82	0–15	I-IV	LRT	40/20	NR	5–8	NR	NR	Yes	243
Navarro et al. 2007 [61]	1993–2002	Spain	RCS/single	61.02	0–15	II-III	CRT	45/25	*a*	4–6	NR	NR	Yes	118
Nisar et al. 2012 [62]	1980–2010	USA	RCS/single	65.20	5.64–11.98	I-IV	LRT	45/25	NR	4–6	NR	*C* ± I	Yes	1862
Pahlman et al. 1993 [63]	1987–1990	Sweden	PCS/multi	NR	NR	I-IV	SRT	25/5	NR	1	NR	NR	NR	1106
Parc et al. 2009 [64]	2000–2004	France	PCS/multi	57.42	NR	I-III	LRT/CRT	NR	NR	NR	NR	*C* ± I	Yes	364
Park et al. 2011 [65]	2004–2006	Korea	PCS/single	62.73	0–10	I-IV	CRT	50/25	c	4–6	NR	NR	NR	218
Park et al. 2016 [66]	2009–2012	Korea	RCS/single	60.24	2.51–4.9	II-III	CRT	50.4/28	*A/c*	6–8	NR	*C* ± I	Yes	166
Rosati et al. 2007 [67]	1997–2007	Italy	RCS/single	50.00	1–12	II-IV	CRT	45/28	*a*	6–8	Lap	C	Yes	46
Salmenkylä et al. 2012 [68]	1995–2002	Finland	PCS/multi	62.23	0–15	I-IV	SRT	25/5	NR	1	NR	*C* ± *E*	Yes	278
Sauer et al. 2004 [4]	1995–2002	Germany	PCS/multi	68.59	0–15	I-IV	CRT	50.4/28	*a*	4–6	NR	*C* ± I	NR	799
Schiffmann et al. 2013 [69]	2000–2009	Germany	RCS/single	66.04	0–16	I-IV	CRT	50.4–55.8/28	*e* + *a*, *d* + *a*, *e* + *c*	NR	NR	NR	Yes	212
Sebag-Montefiore et al. 2009 [6]	1998–2005	UK	PCS/multi	72.67	0–15	I-IV	SRT	25/5	NR	1	NR	C	Yes	1350
Shrikhande et al. 2012 [70]	2003–2011	Indian	RCS/single	66.18	3–8	NR	CRT	50.4/28	*c*	4–6	NR	NR	Yes	68
Sorrentino et al. 2019 [71]	2011–2017	Italy	RCS/single	59.52	0.7–7.9	II-III	CRT	54/25–27	*c*	8	Open	NR	Yes	84
Tabchouri et al. 2020 [72]	2005–2015	France	RCS/multi	63.78	10–15	II-III	CRT/SRT	50/25, 25/5	*c*	7–8, 1	Lap, Open	*C* ± I	Yes	127
Trifunović et al. 2015 [73]	2007–2012	Serbia	RCS/single	68.22	4–15	I-III	SRT	25/5	*NR*	1	Open	NR	NR	107
Tural et al. 2013 [74]	2002–2011	Turkey	RCS/single	61.31	0–15	II	CRT	45–50.4/25–28	*a* + *b*	4–12	NR	NR	NR	137
Valenti et al. 2007 [75]	1995–2004	Spain	RCS/single	67.40	0–16	I-IV	CRT	45–50.4/25	*a*	4–6	NR	C	Yes	273
Valero et al. 2003 [76]	1995–1999	Spain	RCS/single	NR	0–12	II-III	CRT	45/25	*a* + *b*	4–6	NR	NR	Yes	103
Zhan et al. 2019 [77]	2008–2010	China	RCS/single	59.78	0–10	II-III	SRT/CRT	30/15, 50/25	*c*	6–8, 1	Open	*C* ± I	Yes	1197
Zhang et al. 2017 [78]	2008–2014	China	RCS/single	60.40	7.3–9.9	II-III	CRT	50.4/28	*c* + *d*, *a* + *b* + *d*	6–8	Lap, Open	NR	Yes	202

Note: a: 5-fluorouracil, b: Leucovorin, c: capecitabine, d: oxaliplatin, e: Irinotecan, f: S-1, g: Tegafur-uracil; RCS: retrospective cohort study; PCS: prospective controlled study; single: single center; multi: multicenter; NR: not reported; SRT: short-course radiotherapy; LRT: long-course radiotherapy; CRT: chemoradiotherapy; Open: open surgery; Lap: laparoscopic surgery; Rob: robot-assisted surgery; C: clinical manifestation; I: imaging examination; E: endoscopy examination; AL: anastomotic leakage.

**Table 2:** The pooled odds ratio of other postoperative complications.

Studies	Complication	OR (95% CI)	*p* value	Test of heterogeneity		*I* ^*2*^ (%)
				Chi^2^	*p*	
35, 38, 42, 45, 54, 62, 69, 70, 76, 77	Urinary tract infection	1.19 [0.79, 1.79]	0.49	8.97	0.44	0
4, 35, 40, 45, 50, 54, 57, 58, 61, 69, 72, 76, 78, 79	Hemorrhage	1.03 [0.75, 1.40]	0.86	7.97	0.85	0
4, 22, 36, 38, 40, 45, 47, 50, 54, 57, 58, 62, 64-66, 68, 69, 71, 72, 77–79	Ileus	1.21 [0.94, 1.58]	0.14	20.52	0.43	3
4, 35, 36, 38, 40, 42, 44, 46, 48, 50, 53–55, 57, 58, 60, 62, 65, 70, 72, 73, 76, 77, 79	Overall complications	1.15 [0.99, 1.32]	0.06	37.47	0.03	39
35, 36, 38, 55, 58, 68, 72, 73	Reoperation	1.10 [0.77, 1.56]	0.69	7.52	0.38	7
4, 6, 37-40, 44-46, 55, 58, 61, 63, 64, 69, 70, 74, 76, 77	Mortality	1.21 [0.80, 1.84]	0.36	31.38	0.03	43
6, 22, 36–39, 41, 44, 45, 49-52, 54, 58, 59, 61, 63, 67, 70, 72, 73, 76	Defunctioning stoma	3.40 [2.35, 4.91]	<0.01	203.8	<0.01	89

## Data Availability

All data generated or analyzed during this study are included in this article. Further inquiries can be directed to the corresponding author.

## Supplementary Materials

Supplementary Materials
*Supplementary Table 1*. The PRISMA 2020 checklist. *Supplementary Table 2*. Details of postoperative complications in the neoadjuvant radiotherapy and upfront surgery group. *Supplementary Table 3.* The Quality assessment of cohort studies was based on the Newcastle–Ottawa scale. *Supplementary Figure 1*. The effect of neoadjuvant short-course radiotherapy on anastomotic leakage. *Supplementary Figure 2*. Forest plots of anastomotic leakage after neoadjuvant radiotherapy in cohort studies. *Supplementary Figure 3.* Impact of the surgery within 8 weeks after long-course radiotherapy on anastomotic leakage. *Supplementary Figure 4*. Forest plot of perineal wound infection after neoadjuvant radiotherapy. *Supplementary Figure 5.* Forest plot of pelvic abscess after neoadjuvant radiotherapy. *Supplementary Figure 6*. Funnel plot for anastomotic leakage. *Supplementary Figure 7*. Summary of risk of bias judgements for RCTs.
