# Supplementary material for: Effects of Neoadjuvant Radiotherapy on Postoperative Complications in Rectal Cancer: A Meta-Analysis
Source: J Oncol. 2022 Jan 5;2022:8197701. doi: 10.1155/2022/8197701 (PMC8754670; doi:10.1155/2022/8197701)
Supplement: Supplementary Materials — Supplementary Table 1. The PRISMA 2020 checklist. Supplementary Table 2. Details of postoperative complications in the neoadjuvant radiotherapy and upfront surgery group. Supplementary Table 3. The Quality assessment of cohort studies was based on the Newcastle–Ottawa scale. Supplementary Figure 1. The effect of neoadjuvant short-course radiotherapy on anastomotic leakage. Supplementary Figure 2. Forest plots of anastomotic leakage after neoadjuvant radiotherapy in cohort studies. Supplementary Figure 3. Impact of the surgery within 8 weeks after long-course radiotherapy on anastomotic leakage. Supplementary Figure 4. Forest plot of perineal wound infection after neoadjuvant radiotherapy. Supplementary Figure 5. Forest plot of pelvic abscess after neoadjuvant radiotherapy. Supplementary Figure 6. Funnel plot for anastomotic leakage. Supplementary Figure 7. Summary of risk of bias judgements for RCTs. [file 8197701.f1.zip › 8197701.f1/Figure S7.pdf]

Study ID	Outcome	Randomization process	Deviations from intended intervention	Missing outcome data	Measurement of the outcome	Selection of the reported result	Overall
Cedermark et al. 1995	Anastomotic Leakage	+	+	+	+	+	+
Fan et al. 2015	Anastomotic Leakage	+	?	+	+	+	!
Marijnen et al. 2002	Anastomotic Leakage	+	+	+	+	+	+
MRCRCWP. 1996	Anastomotic Leakage	+	+	+	+	+	+
Pahlman et al. 1993	Anastomotic Leakage	+	?	+	+	+	!
Parc et al. 2009	Anastomotic Leakage	?	?	+	+	+	!
park et al. 2011	Anastomotic Leakage	+	+	+	+	+	+
Salmenkylä et al. 2012	Anastomotic Leakage	+	?	+	+	+	!
Sauer et al. 2004	Anastomotic Leakage	+	+	+	+	+	+
Sebag-Montefiore et al. 2009	Anastomotic Leakage	+	+	+	+	+	+

- **+** **Low risk**
- **?** **Some concerns**
- **?** **High risk**
