# Supplementary material for: Effects of Neoadjuvant Radiotherapy on Postoperative Complications in Rectal Cancer: A Meta-Analysis
Source: J Oncol. 2022 Jan 5;2022:8197701. doi: 10.1155/2022/8197701 (PMC8754670; doi:10.1155/2022/8197701)
Supplement: Supplementary Materials — Supplementary Table 1. The PRISMA 2020 checklist. Supplementary Table 2. Details of postoperative complications in the neoadjuvant radiotherapy and upfront surgery group. Supplementary Table 3. The Quality assessment of cohort studies was based on the Newcastle–Ottawa scale. Supplementary Figure 1. The effect of neoadjuvant short-course radiotherapy on anastomotic leakage. Supplementary Figure 2. Forest plots of anastomotic leakage after neoadjuvant radiotherapy in cohort studies. Supplementary Figure 3. Impact of the surgery within 8 weeks after long-course radiotherapy on anastomotic leakage. Supplementary Figure 4. Forest plot of perineal wound infection after neoadjuvant radiotherapy. Supplementary Figure 5. Forest plot of pelvic abscess after neoadjuvant radiotherapy. Supplementary Figure 6. Funnel plot for anastomotic leakage. Supplementary Figure 7. Summary of risk of bias judgements for RCTs. [file 8197701.f1.zip › 8197701.f1/Table S1-S3.pdf]

Table S1 The PRISMA 2020 checklist

Section and Topic	Item #	Checklist item	Location where item is reported
**TITLE**			
Title	1	Identify the report as a systematic review.	1
**ABSTRACT**			
Abstract	2	See the PRISMA 2020 for Abstracts checklist.	2
**INTRODUCTION**			
Rationale	3	Describe the rationale for the review in the context of existing knowledge.	3
Objectives	4	Provide an explicit statement of the objective(s) or question(s) the review addresses.	4
**METHODS**			
Eligibility criteria	5	Specify the inclusion and exclusion criteria for the review and how studies were grouped for the syntheses.	4
Information sources	6	Specify all databases, registers, websites, organisations, reference lists and other sources searched or consulted to identify studies. Specify the date when each source was last searched or consulted.	4
Search strategy	7	Present the full search strategies for all databases, registers and websites, including any filters and limits used.	4,5
Selection process	8	Specify the methods used to decide whether a study met the inclusion criteria of the review, including how many reviewers screened each record and each report retrieved, whether they worked independently, and if applicable, details of automation tools used in the process.	5
Data collection process	9	Specify the methods used to collect data from reports, including how many reviewers collected data from each report, whether they worked independently, any processes for obtaining or confirming data from study investigators, and if applicable, details of automation tools used in the process.	5,6
Data items	10a	List and define all outcomes for which data were sought. Specify whether all results that were compatible with each outcome domain in each study were sought (e.g. for all measures, time points, analyses), and if not, the methods used to decide which results to collect.	6
	10b	List and define all other variables for which data were sought (e.g. participant and intervention characteristics, funding sources). Describe any assumptions made about any missing or unclear information.	6
Study risk of bias assessment	11	Specify the methods used to assess risk of bias in the included studies, including details of the tool(s) used, how many reviewers assessed each study and whether they worked independently, and if applicable, details of automation tools used in the process.	6
Effect measures	12	Specify for each outcome the effect measure(s) (e.g. risk ratio, mean difference) used in the synthesis or presentation of results.	7
Synthesis methods	13a	Describe the processes used to decide which studies were eligible for each synthesis (e.g. tabulating the study intervention characteristics and comparing against the planned groups for each synthesis (item #5)).	7
	13b	Describe any methods required to prepare the data for presentation or synthesis, such as handling of missing summary statistics, or data conversions.	7
	13c	Describe any methods used to tabulate or visually display results of individual studies and syntheses.	7
	13d	Describe any methods used to synthesize results and provide a rationale for the choice(s). If meta-analysis was performed, describe the model(s), method(s) to identify the presence and extent of statistical heterogeneity, and software package(s) used.	7
	13e	Describe any methods used to explore possible causes of heterogeneity among study results (e.g. subgroup analysis, meta-regression).	7
	13f	Describe any sensitivity analyses conducted to assess robustness of the synthesized results.	7
Reporting bias assessment	14	Describe any methods used to assess risk of bias due to missing results in a synthesis (arising from reporting biases).	7
Certainty assessment	15	Describe any methods used to assess certainty (or confidence) in the body of evidence for an outcome.	7
**RESULTS**			

Section and Topic	Item #	Checklist item	Location where item is reported
Study selection	16a	Describe the results of the search and selection process, from the number of records identified in the search to the number of studies included in the review, ideally using a flow diagram.	8
	16b	Cite studies that might appear to meet the inclusion criteria, but which were excluded, and explain why they were excluded.	8
Study characteristics	17	Cite each included study and present its characteristics.	8
Risk of bias in studies	18	Present assessments of risk of bias for each included study.	8
Results of individual studies	19	For all outcomes, present, for each study: (a) summary statistics for each group (where appropriate) and (b) an effect estimate and its precision (e.g. confidence/credible interval), ideally using structured tables or plots.	9,10,11
Results of syntheses	20a	For each synthesis, briefly summarise the characteristics and risk of bias among contributing studies.	9,10,11
	20b	Present results of all statistical syntheses conducted. If meta-analysis was done, present for each the summary estimate and its precision (e.g. confidence/credible interval) and measures of statistical heterogeneity. If comparing groups, describe the direction of the effect.	9,10,11
	20c	Present results of all investigations of possible causes of heterogeneity among study results.	9,10,11
	20d	Present results of all sensitivity analyses conducted to assess the robustness of the synthesized results.	
Reporting biases	21	Present assessments of risk of bias due to missing results (arising from reporting biases) for each synthesis assessed.	11
Certainty of evidence	22	Present assessments of certainty (or confidence) in the body of evidence for each outcome assessed.	
**DISCUSSION**			
Discussion	23a	Provide a general interpretation of the results in the context of other evidence.	11
	23b	Discuss any limitations of the evidence included in the review.	15,16
	23c	Discuss any limitations of the review processes used.	15,16
	23d	Discuss implications of the results for practice, policy, and future research.	16
**OTHER INFORMATION**			
Registration and protocol	24a	Provide registration information for the review, including register name and registration number, or state that the review was not registered.	
	24b	Indicate where the review protocol can be accessed, or state that a protocol was not prepared.	
	24c	Describe and explain any amendments to information provided at registration or in the protocol.	
Support	25	Describe sources of financial or non-financial support for the review, and the role of the funders or sponsors in the review.	17
Competing interests	26	Declare any competing interests of review authors.	17
Availability of data, code and other materials	27	Report which of the following are publicly available and where they can be found: template data collection forms; data extracted from included studies; data used for all analyses; analytic code; any other materials used in the review.	17

From: Page MJ, McKenzie JE, Bossuyt PM, Boutron I, Hoffmann TC, Mulrow CD, et al. The PRISMA 2020 statement: an updated guideline for reporting systematic reviews. BMJ 2021;372:n71. doi: 10.1136/bmj.n71

For more information, visit: http://www.prisma-statement.org/

Table S2 Details of postoperative complications in the neoadjuvant radiotherapy and upfront surgery group

Study	Neoadjuvant radiotherapy group										Upfront surgery group									
	AL	Wound infection	Perineal wound infection	Pelvic abscess	Urinary tract infection	Hemorrhage	Ileus	Reoperation	Overall complications	Mortality	AL	Wound infection	Perineal wound infection	Pelvic abscess	Urinary tract infection	Hemorrhage	Ileus	Reoperation	Overall complications	Mortality
Akgun et al. 2017[27]	3/101	7/177	NR	1/177	3/177	2/177	NR	2/177	29/177	NR	0/109	4/159	NR	1/159	4/159	1/159	NR	1/159	23/159	NR
Akiyoshi et al. 2009[28]	0/14	3/20	NR	NR	NR	NR	1/20	0/20	4/20	NR	3/95	3/105	NR	NR	NR	NR	0/105	3/105	12/105	NR
Bhangu et al. 2010[29]	1/22	NR	NR	1/38	NR	NR	NR	NR	NR	2/38	3/41	NR	NR	3/46	NR	NR	NR	NR	NR	1/46
Bosch et al. 2017[30]	0/17	13/61	7/44	5/61	11/61	NR	4/61	11/61	32/61	1/61	3/38	9/84	6/46	3/84	7/84	NR	2/84	14/84	33/84	2/84
Buie et al. 2005[31]	3/36	NR	NR	6/60	NR	NR	NR	NR	NR	1/60	6/150	NR	NR	3/186	NR	NR	NR	NR	NR	5/186
Cedermark et al. 1995[32]	10/152	61/389	NR	NR	NR	5/389	8/389	NR	112/389	35/389	15/153	37/402	NR	NR	NR	4/402	13/402	NR	81/402	7/402
Chang et al. 2014[33]	27/360	NR	NR	NR	NR	NR	NR	NR	NR	NR	29/360	NR	NR	NR	NR	NR	NR	NR	NR	NR
Cheung et al. 2008[34]	2/38	2/52	0/14	1/52	2/52	NR	NR	NR	18/52	NR	2/99	10/138	4/39	1/138	2/138	NR	NR	NR	47/138	NR
Creavin et al. 2019[35]	31/252	NR	NR	NR	NR	NR	NR	NR	NR	NR	19/250	NR	NR	NR	NR	NR	NR	NR	NR	NR
Denost et al. 2012[36]	37/292	NR	NR	NR	NR	NR	NR	NR	107/292	1/292	14/130	NR	NR	NR	NR	NR	NR	NR	38/130	1/130
Doeksen et al. 2007[37]	11/28	4/39	NR	NR	2/39	1/39	5/39	NR	NR	10/39	10/54	4/65	NR	NR	2/65	2/65	3/65	NR	NR	5/65
Enker et al. 1999[38]	6/150	8/150	NR	6/150	NR	NR	NR	NR	26/150	0/150	21/531	37/531	NR	5/531	NR	NR	NR	NR	127/531	6/531
Fan et al. 2015[39]	2/63	4/90	NR	NR	NR	NR	1/90	NR	NR	NR	8/67	4/94	NR	NR	NR	NR	1/94	NR	NR	NR
Frouws et al. 2017 [26]	358/3497	NR	NR	NR	NR	NR	NR	NR	NR	NR	60/790	NR	NR	NR	NR	NR	NR	NR	NR	NR
Fucini et al. 2010[40]	4/27	NR	NR	NR	NR	NR	NR	NR	21/47	NR	2/21	NR	NR	NR	NR	NR	NR	NR	14/33	NR
Garlipp et al. 2010[41]	84/676	NR	NR	NR	NR	NR	NR	NR	NR	NR	179/1409	NR	NR	NR	NR	NR	NR	NR	NR	NR
Hassan et al. 2008 [19]	2/39	NR	NR	3/39	NR	NR	0/39	NR	NR	NR	0/87	NR	NR	0/87	NR	NR	1/87	NR	NR	NR
Kang et al. 2012[42]	0/36	1/47	NR	NR	NR	1/47	2/47	NR	6/47	NR	4/36	1/47	NR	NR	NR	1/47	5/47	NR	11/47	NR
Kao et al. 2010[43]	7/52	NR	NR	6/69	NR	NR	NR	NR	NR	NR	2/34	NR	NR	1/67	NR	NR	NR	NR	NR	NR
Kim et al. 2010[44]	11/285	NR	NR	NR	NR	NR	NR	NR	37/285	NR	13/418	NR	NR	NR	NR	NR	NR	NR	42/418	NR
Kim et al. 2018[45]	2/28	NR	NR	NR	NR	NR	NR	NR	NR	NR	6/51	NR	NR	NR	NR	NR	NR	NR	NR	NR
Korkolis et al. 2006[46]	2/40	3/40	NR	NR	2/40	1/40	2/40	NR	16/40	NR	3/37	2/45	NR	NR	3/45	1/45	2/45	NR	19/45	NR
Lim et al. 2010[47]	2/9	8/58	NR	NR	NR	NR	NR	3/58	17/58	0/58	2/37	14/122	NR	NR	NR	NR	NR	9/122	51/122	1/122
Lyall et al. 2006[48]	10/37	NR	NR	NR	NR	NR	NR	NR	NR	NR	2/50	NR	NR	NR	NR	NR	NR	NR	NR	NR
Madbouly et al. 2015[49]	6/48	2/55	NR	NR	NR	0/55	4/55	NR	9/55	NR	1/29	2/41	NR	NR	NR	1/41	3/41	NR	7/41	NR
Marijnen et al. 2002[50]	49/439	104/695	NR	31/695	NR	23/695	22/695	103/695	336/695	28/695	56/465	84/719	NR	20/719	NR	29/719	10/719	98/719	297/719	24/719
Martel et al. 2008[51]	3/54	NR	NR	NR	NR	NR	NR	NR	NR	NR	11/166	NR	NR	NR	NR	NR	NR	NR	NR	NR
Milgrom et al. 2014[52]	3/253	29/310	6/53	11/310	NR	NR	NR	NR	77/310	NR	3/130	21/151	2/19	1/151	NR	NR	NR	NR	40/151	NR

MRCRCWP. 1996[53]	9/38	28/139	10/70	NR	NR	5/139	NR	NR	NR	5/139	10/38	23/140	5/75	NR	NR	12/140	NR	NR	NR	10/140
Navarro et al. 2007[54]	5/47	5/61	5/12	1/61	2/61	NR	5/61	NR	29/61	NR	0/38	3/57	3/18	1/57	2/57	NR	2/57	NR	18/57	NR
Nisar et al. 2012[55]	42/524	NR	NR	NR	NR	NR	NR	NR	NR	6/524	76/1338	NR	NR	NR	NR	NR	NR	NR	NR	24/1338
Pahlman et al. 1993[56]	26/243	88/547	63/304	NR	NR	NR	27/547	NR	NR	22/547	17/227	57/559	29/332	NR	NR	NR	20/559	NR	NR	15/559
Parc et al. 2009[57]	11/211	7/211	NR	15/211	NR	NR	7/211	NR	63/211	NR	8/153	4/153	NR	8/153	NR	NR	13/153	NR	54/153	NR
Park et al. 2011[58]	2/84	NR	NR	2/105	NR	NR	5/105	NR	NR	NR	5/81	NR	NR	0/113	NR	NR	5/113	NR	NR	NR
Park et al. 2016[59]	5/54	NR	NR	NR	NR	NR	NR	NR	NR	NR	4/56	NR	NR	NR	NR	NR	NR	NR	NR	NR
Rosati et al. 2007[60]	2/18	NR	NR	NR	NR	NR	0/20	4/20	NR	NR	7/26	NR	NR	NR	NR	NR	1/26	9/26	NR	NR
Salmenkylä et al. 2012[61]	23/84	12/130	15/39	3/130	11/130	4/130	8/130	NR	NR	5/130	21/102	4/148	7/44	1/148	12/148	4/148	13/148	NR	NR	4/148
Sauer et al. 2004[4]	46/286	NR	NR	NR	NR	12/415	8/415	NR	149/415	3/415	46/273	NR	NR	NR	NR	8/384	4/384	NR	131/384	5/384
Schiffmann et al. 2013[62]	11/41	22/106	13/31	NR	3/106	NR	NR	NR	57/106	0/106	4/43	10/106	6/32	NR	10/106	NR	NR	NR	54/106	2/106
Sebag-Montefiore et al. 2009[6]	32/383	NR	70/202	NR	NR	NR	NR	NR	NR	12/674	26/409	NR	44/202	NR	NR	NR	NR	NR	NR	15/676
Shrikhande et al. 2012[63]	2/45	3/45	NR	NR	NR	NR	1/45	NR	NR	NR	1/23	1/23	NR	NR	NR	NR	0/23	NR	NR	NR
Sorrentino et al. 2019[64]	3/37	NR	NR	8/42	NR	3/42	1/42	5/42	12/42	NR	4/41	NR	NR	3/42	NR	1/42	0/42	1/42	8/42	NR
Tabchouri et al. 2020[65]	5/48	NR	NR	NR	NR	NR	NR	4/48	29/48	NR	2/79	NR	NR	NR	NR	NR	NR	0/79	24/79	NR
Trifunović et al. 2015[66]	12/51	14/51	NR	NR	NR	NR	NR	NR	NR	5/51	5/56	8/56	NR	NR	NR	NR	NR	NR	NR	1/56
Tural et al. 2013[67]	1/36	NR	NR	3/58	NR	NR	NR	NR	NR	NR	2/47	NR	NR	3/79	NR	NR	NR	NR	NR	NR
Valenti et al. 2007[68]	5/121	15/170	NR	8/170	15/170	7/170	NR	NR	39/170	1/170	3/80	7/103	NR	5/170	10/170	4/170	NR	NR	33/170	1/103
Valero et al. 2003[69]	3/40	7/53	5/11	NR	1/53	NR	2/53	NR	20/53	1/53	1/30	8/50	5/19	NR	0/50	NR	2/50	NR	22/50	0/50
Zhan et al. 2019[70]	25/387	NR	42/218	NR	NR	20/605	25/605	NR	NR	NR	23/507	NR	8/85	NR	NR	14/592	14/592	NR	NR	NR
Zhang et al. 2017[71]	6/96	4/101	NR	NR	NR	2/101	7/101	NR	28/101	NR	4/99	5/101	NR	NR	NR	3/101	6/101	NR	29/101	NR

Notes: AL: Anastomotic leakage; NR: Not report

Table S3 The Quality assessment of cohort studies was based on the Newcastle–Ottawa scale

Study	Representativeness of the exposed cohort	Selection of the non-exposed cohort	Ascertainment of exposure	Demonstration that outcome of interest was not present at start of study	Comparability of cohorts on the basis of the design or analysis	Assessment of outcome	Was follow-up long enough for outcomes to occur	Adequacy of follow-up of cohorts	Quality score
Akgun et al. 2017[27]	1	1	1	1	1	1	1	1	8
Akiyoshi et al. 2009[28]	1	1	1	1	1	0	0	0	5
Bhangu et al. 2010[29]	1	1	1	1	1	1	0	0	6
Bosch et al. 2017[30]	1	1	1	1	1	0	0	0	5
Buie et al. 2005[31]	1	1	1	1	1	1	0	0	6
Chang et al. 2014[33]	1	1	1	0	1	1	0	0	5
Cheung et al. 2008[34]	1	1	1	1	1	1	1	0	7
Creavin et al. 2019[35]	1	1	1	1	0	1	0	0	5
Denost et al. 2012[36]	1	1	1	1	1	1	0	0	6
Doeksen et al. 2007[37]	1	1	1	1	1	1	0	1	7
Enker et al. 1999[38]	1	1	1	1	1	1	1	0	7
Frouws et al. 2017 [26]	1	1	1	1	1	1	0	0	6
Fucini et al. 2010[40]	1	1	1	1	1	0	1	0	6
Garlipp et al. 2010[41]	1	1	1	1	1	1	0	0	6
Hassan et al. 2008 [19]	1	1	1	1	1	1	1	0	7
Kang et al. 2012[42]	1	1	1	1	1	0	1	1	7
Kao et al. 2010[43]	1	1	1	1	1	0	1	1	7
Kim et al. 2010[44]	1	1	1	1	1	0	1	1	7
Kim et al. 2018[45]	1	1	1	1	1	0	1	0	6
Korkolis et al. 2006[46]	1	1	1	1	1	1	0	0	6
Lim et al. 2010[47]	1	1	1	1	1	1	1	0	7
Lyall et al. 2006[48]	1	1	1	1	1	0	0	0	5
Madbouly et al. 2015[49]	1	1	1	1	1	0	1	0	6
Martel et al. 2008[51]	1	1	1	1	1	1	0	0	6
Milgrom et al. 2014[52]	1	1	1	1	1	1	0	0	6
Navarro et al. 2007[54]	1	1	1	1	1	0	1	0	6
Nisar et al. 2012[55]	1	1	1	1	1	1	0	0	6
Park et al. 2016[59]	1	1	1	1	1	1	0	0	6
Rosati et al. 2007[60]	1	1	1	1	1	1	0	0	6
Schiffmann et al. 2013[62]	1	1	1	1	1	1	0	0	6
Shrikhande et al. 2012[63]	1	1	1	1	1	0	0	0	5
Sorrentino et al. 2019[64]	1	1	1	1	1	0	1	0	6
Tabchouri et al. 2020[65]	1	1	1	1	1	1	0	0	6
Trifunović et al. 2015[66]	1	1	1	1	1	0	0	0	5
Tural et al. 2013[67]	1	1	1	1	1	1	1	0	6

Valenti et al. 2007[68]	1	1	1	1	1	1	0	0	6
Valero et al. 2003[69]	1	1	1	1	1	0	0	0	5
Zhan et al. 2019[70]	1	1	1	1	1	1	0	0	6
Zhang et al. 2017[71]	1	1	1	1	1	0	1	0	6
