# Supplementary figures and images for: Effects of Neoadjuvant Radiotherapy on Postoperative Complications in Rectal Cancer: A Meta-Analysis
Source: J Oncol. 2022 Jan 5;2022:8197701. doi: 10.1155/2022/8197701 (PMC8754670; doi:10.1155/2022/8197701)

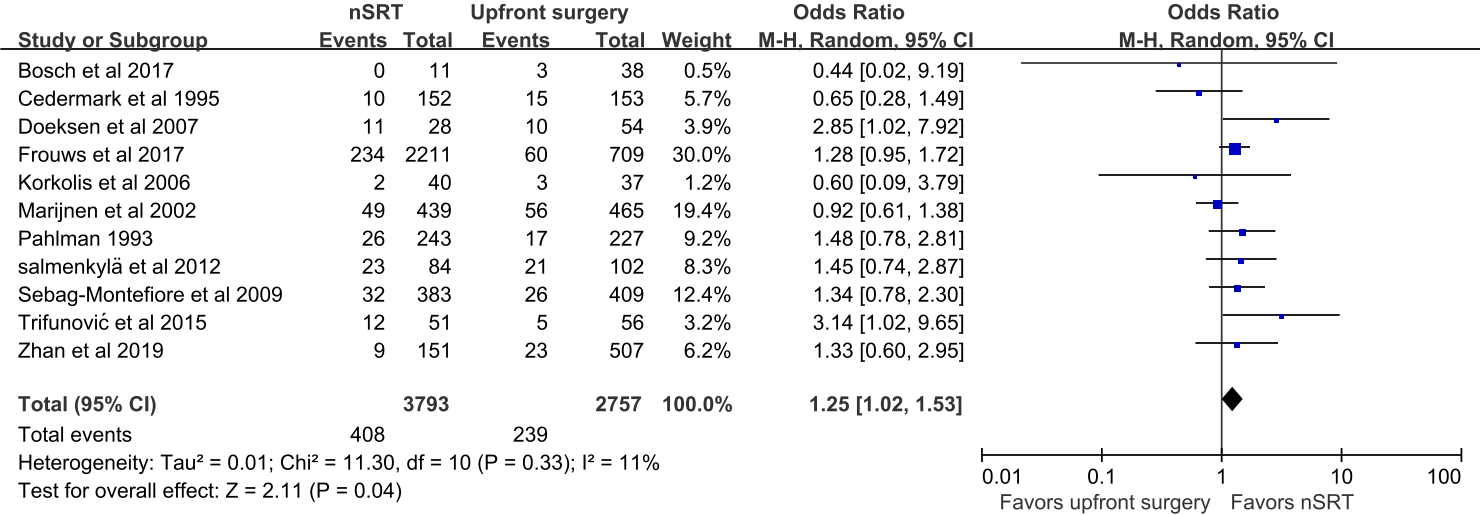

Supplement: Supplementary Materials — Supplementary Table 1. The PRISMA 2020 checklist. Supplementary Table 2. Details of postoperative complications in the neoadjuvant radiotherapy and upfront surgery group. Supplementary Table 3. The Quality assessment of cohort studies was based on the Newcastle–Ottawa scale. Supplementary Figure 1. The effect of neoadjuvant short-course radiotherapy on anastomotic leakage. Supplementary Figure 2. Forest plots of anastomotic leakage after neoadjuvant radiotherapy in cohort studies. Supplementary Figure 3. Impact of the surgery within 8 weeks after long-course radiotherapy on anastomotic leakage. Supplementary Figure 4. Forest plot of perineal wound infection after neoadjuvant radiotherapy. Supplementary Figure 5. Forest plot of pelvic abscess after neoadjuvant radiotherapy. Supplementary Figure 6. Funnel plot for anastomotic leakage. Supplementary Figure 7. Summary of risk of bias judgements for RCTs. [file 8197701.f1.zip › 8197701.f1/Figure S1.pdf]

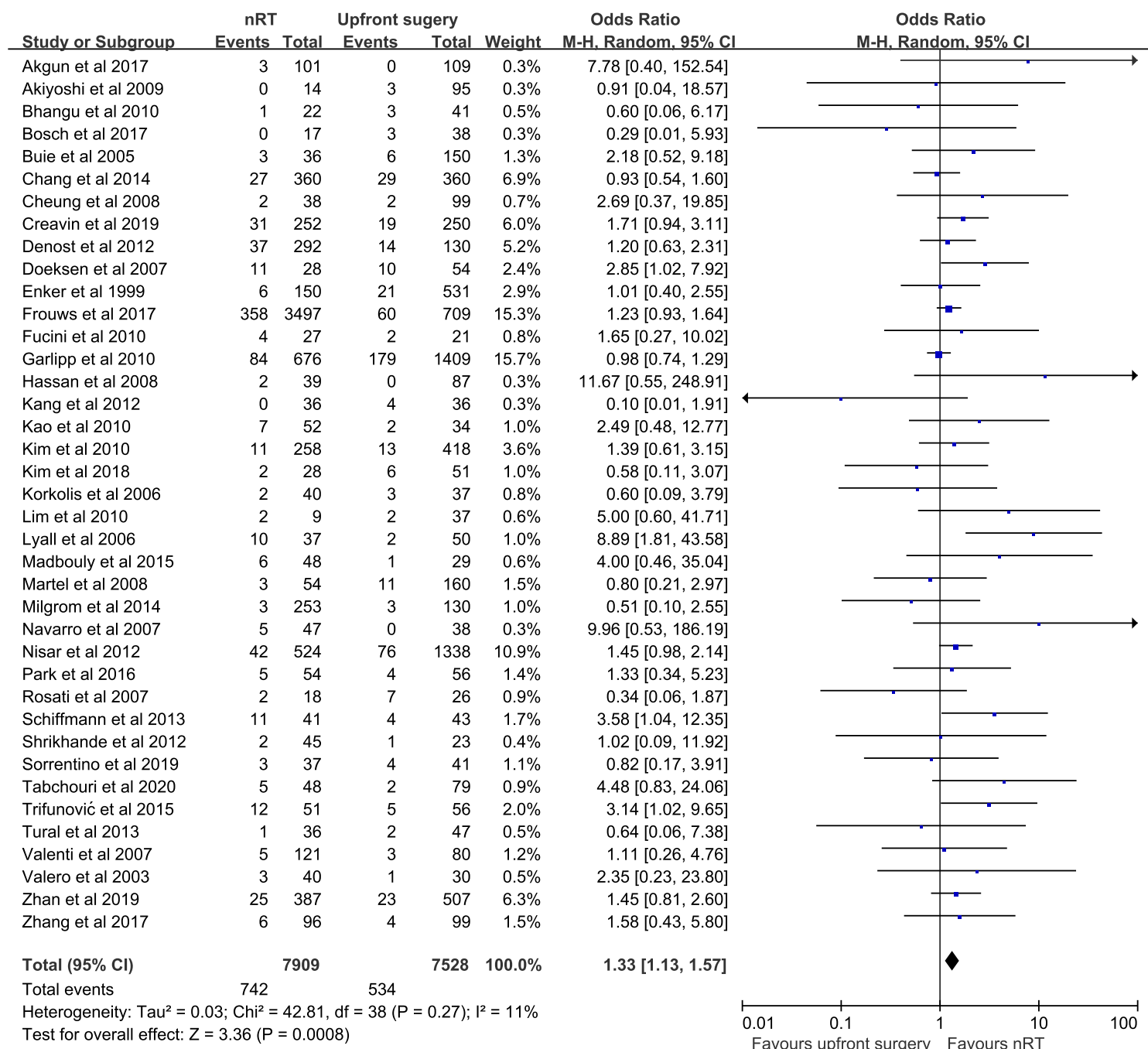

Supplement: Supplementary Materials — Supplementary Table 1. The PRISMA 2020 checklist. Supplementary Table 2. Details of postoperative complications in the neoadjuvant radiotherapy and upfront surgery group. Supplementary Table 3. The Quality assessment of cohort studies was based on the Newcastle–Ottawa scale. Supplementary Figure 1. The effect of neoadjuvant short-course radiotherapy on anastomotic leakage. Supplementary Figure 2. Forest plots of anastomotic leakage after neoadjuvant radiotherapy in cohort studies. Supplementary Figure 3. Impact of the surgery within 8 weeks after long-course radiotherapy on anastomotic leakage. Supplementary Figure 4. Forest plot of perineal wound infection after neoadjuvant radiotherapy. Supplementary Figure 5. Forest plot of pelvic abscess after neoadjuvant radiotherapy. Supplementary Figure 6. Funnel plot for anastomotic leakage. Supplementary Figure 7. Summary of risk of bias judgements for RCTs. [file 8197701.f1.zip › 8197701.f1/Figure S2.pdf]

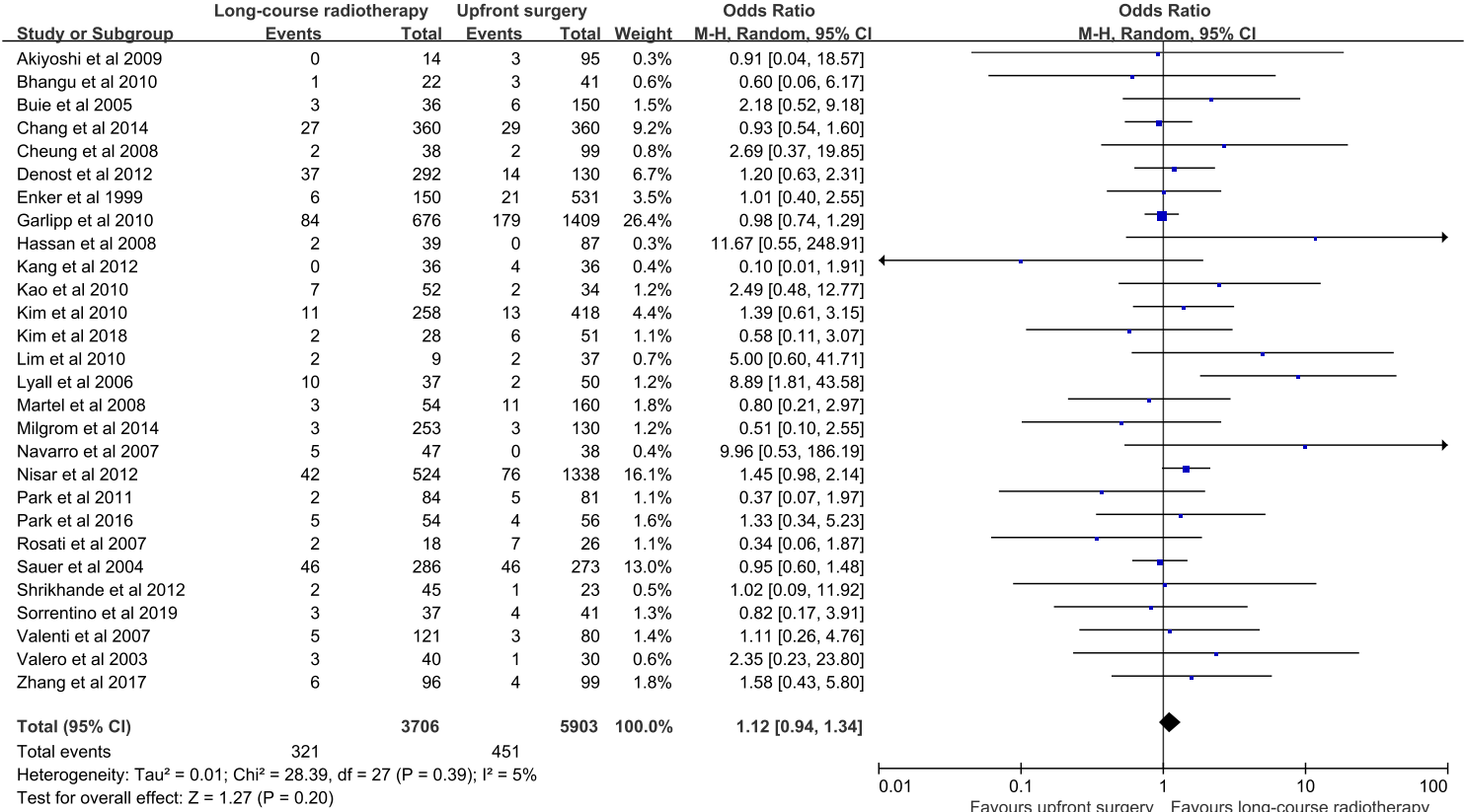

Supplement: Supplementary Materials — Supplementary Table 1. The PRISMA 2020 checklist. Supplementary Table 2. Details of postoperative complications in the neoadjuvant radiotherapy and upfront surgery group. Supplementary Table 3. The Quality assessment of cohort studies was based on the Newcastle–Ottawa scale. Supplementary Figure 1. The effect of neoadjuvant short-course radiotherapy on anastomotic leakage. Supplementary Figure 2. Forest plots of anastomotic leakage after neoadjuvant radiotherapy in cohort studies. Supplementary Figure 3. Impact of the surgery within 8 weeks after long-course radiotherapy on anastomotic leakage. Supplementary Figure 4. Forest plot of perineal wound infection after neoadjuvant radiotherapy. Supplementary Figure 5. Forest plot of pelvic abscess after neoadjuvant radiotherapy. Supplementary Figure 6. Funnel plot for anastomotic leakage. Supplementary Figure 7. Summary of risk of bias judgements for RCTs. [file 8197701.f1.zip › 8197701.f1/Figure S3.pdf]

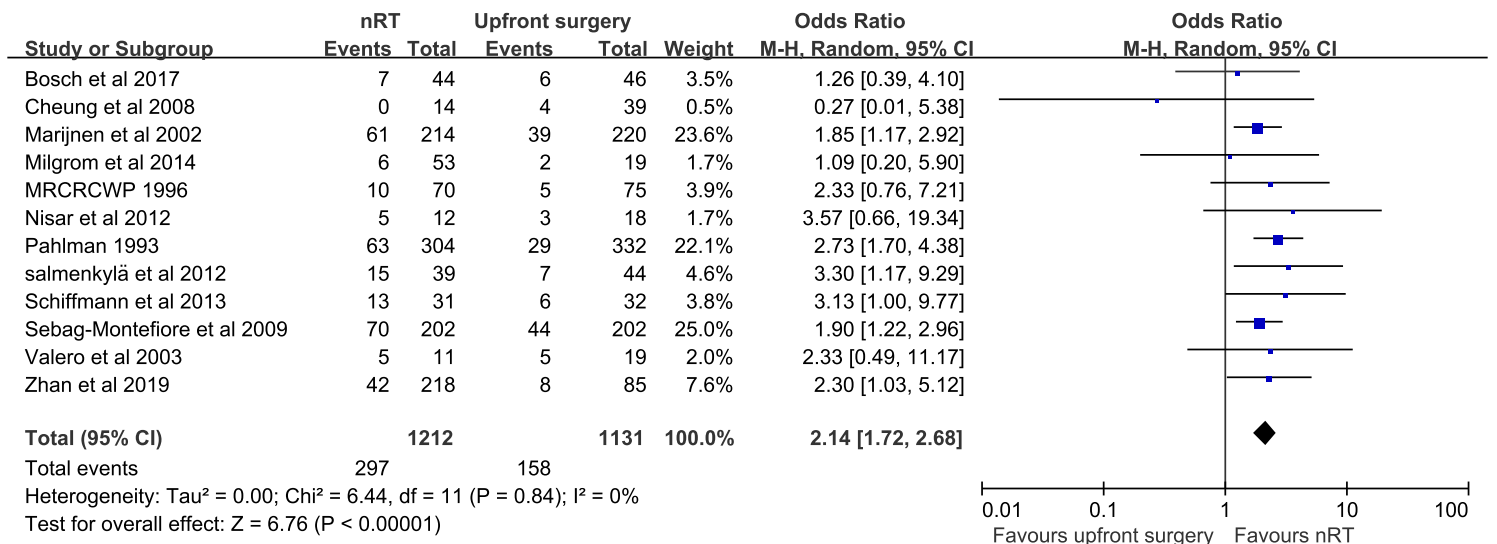

Supplement: Supplementary Materials — Supplementary Table 1. The PRISMA 2020 checklist. Supplementary Table 2. Details of postoperative complications in the neoadjuvant radiotherapy and upfront surgery group. Supplementary Table 3. The Quality assessment of cohort studies was based on the Newcastle–Ottawa scale. Supplementary Figure 1. The effect of neoadjuvant short-course radiotherapy on anastomotic leakage. Supplementary Figure 2. Forest plots of anastomotic leakage after neoadjuvant radiotherapy in cohort studies. Supplementary Figure 3. Impact of the surgery within 8 weeks after long-course radiotherapy on anastomotic leakage. Supplementary Figure 4. Forest plot of perineal wound infection after neoadjuvant radiotherapy. Supplementary Figure 5. Forest plot of pelvic abscess after neoadjuvant radiotherapy. Supplementary Figure 6. Funnel plot for anastomotic leakage. Supplementary Figure 7. Summary of risk of bias judgements for RCTs. [file 8197701.f1.zip › 8197701.f1/Figure S4.pdf]

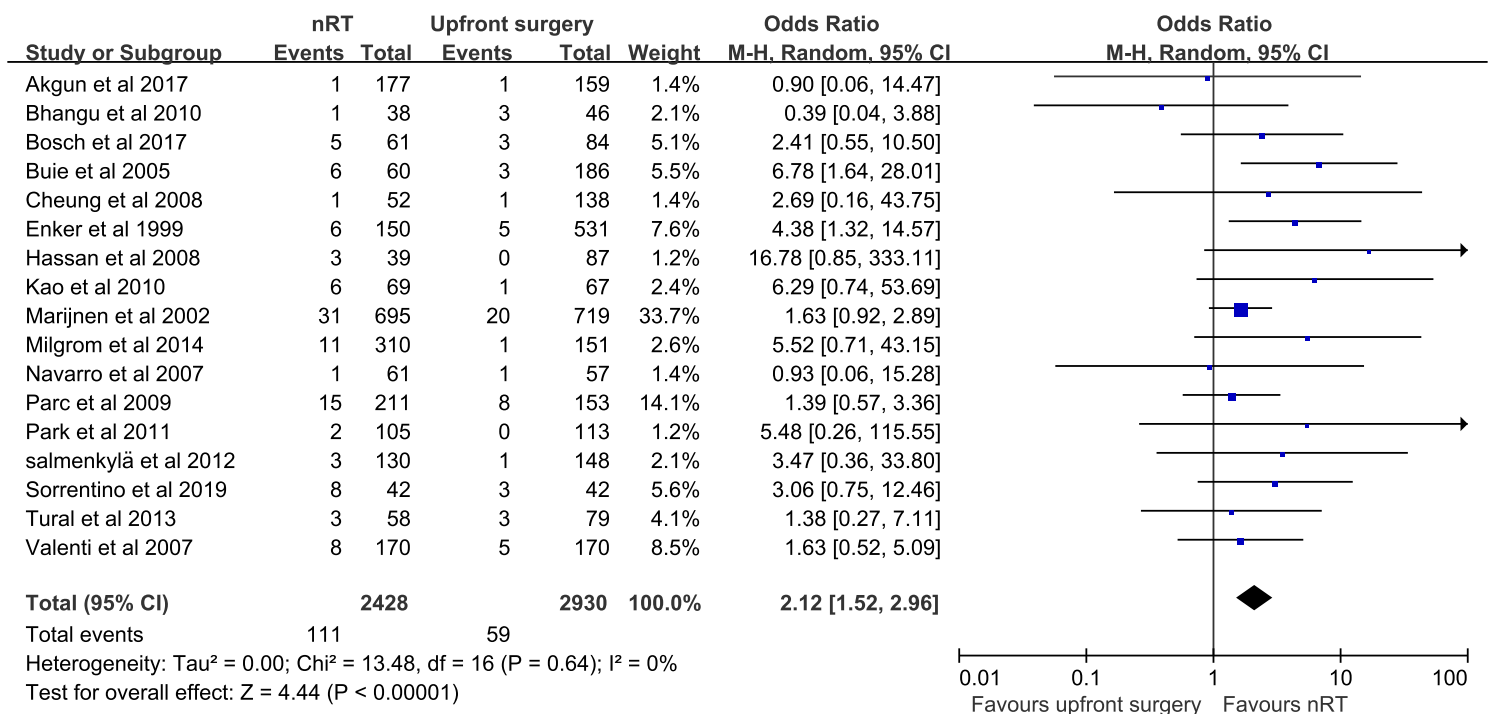

Supplement: Supplementary Materials — Supplementary Table 1. The PRISMA 2020 checklist. Supplementary Table 2. Details of postoperative complications in the neoadjuvant radiotherapy and upfront surgery group. Supplementary Table 3. The Quality assessment of cohort studies was based on the Newcastle–Ottawa scale. Supplementary Figure 1. The effect of neoadjuvant short-course radiotherapy on anastomotic leakage. Supplementary Figure 2. Forest plots of anastomotic leakage after neoadjuvant radiotherapy in cohort studies. Supplementary Figure 3. Impact of the surgery within 8 weeks after long-course radiotherapy on anastomotic leakage. Supplementary Figure 4. Forest plot of perineal wound infection after neoadjuvant radiotherapy. Supplementary Figure 5. Forest plot of pelvic abscess after neoadjuvant radiotherapy. Supplementary Figure 6. Funnel plot for anastomotic leakage. Supplementary Figure 7. Summary of risk of bias judgements for RCTs. [file 8197701.f1.zip › 8197701.f1/Figure S5.pdf]

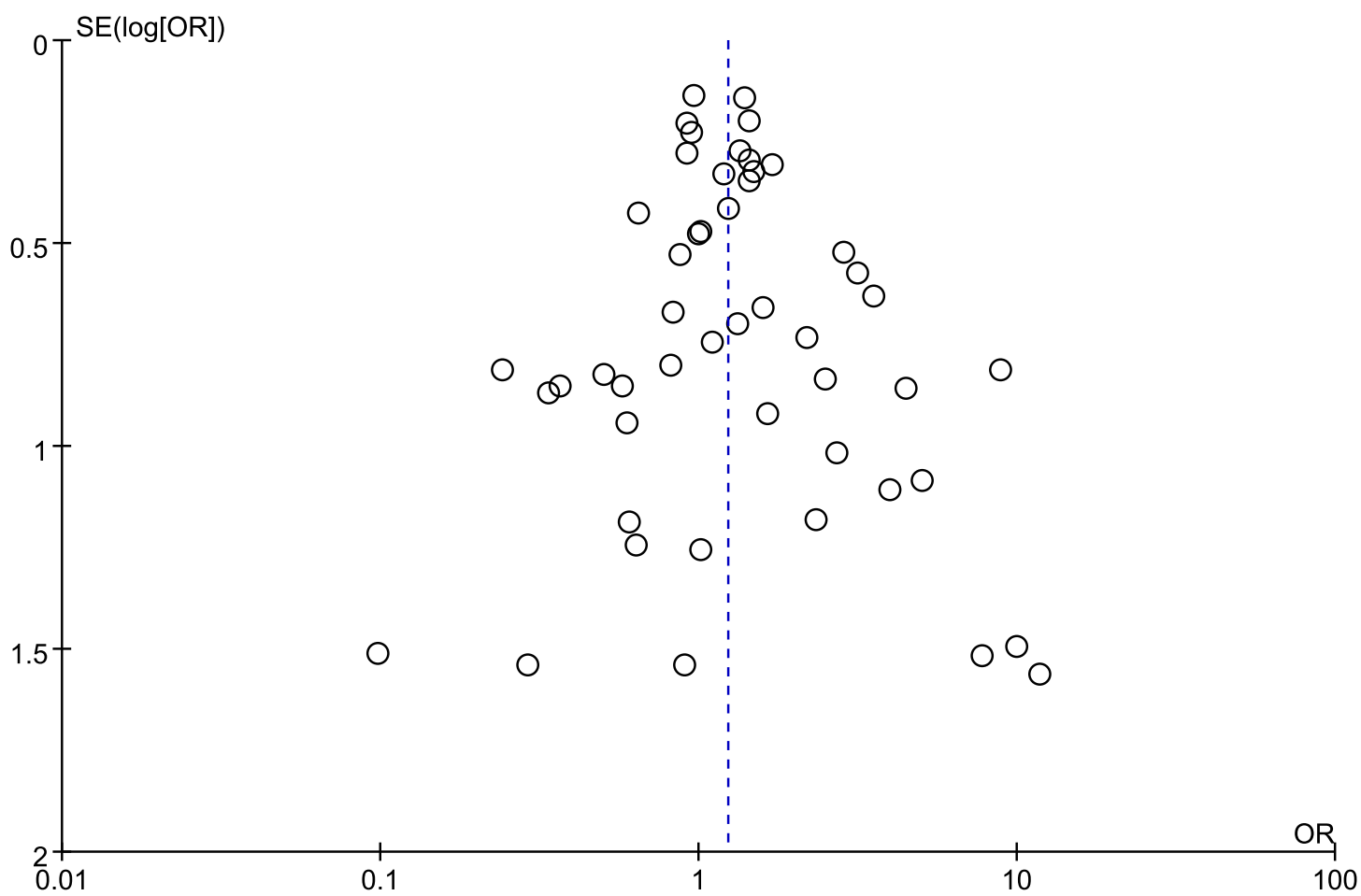

Supplement: Supplementary Materials — Supplementary Table 1. The PRISMA 2020 checklist. Supplementary Table 2. Details of postoperative complications in the neoadjuvant radiotherapy and upfront surgery group. Supplementary Table 3. The Quality assessment of cohort studies was based on the Newcastle–Ottawa scale. Supplementary Figure 1. The effect of neoadjuvant short-course radiotherapy on anastomotic leakage. Supplementary Figure 2. Forest plots of anastomotic leakage after neoadjuvant radiotherapy in cohort studies. Supplementary Figure 3. Impact of the surgery within 8 weeks after long-course radiotherapy on anastomotic leakage. Supplementary Figure 4. Forest plot of perineal wound infection after neoadjuvant radiotherapy. Supplementary Figure 5. Forest plot of pelvic abscess after neoadjuvant radiotherapy. Supplementary Figure 6. Funnel plot for anastomotic leakage. Supplementary Figure 7. Summary of risk of bias judgements for RCTs. [file 8197701.f1.zip › 8197701.f1/Figure S6.pdf]
